# 初诊多发性骨髓瘤原始幼稚浆细胞负荷量的预后价值

**DOI:** 10.3760/cma.j.issn.0253-2727.2022.01.014

**Published:** 2022-01

**Authors:** 玉龙 李, 杰 时, 凤珍 刘, 晓燕 董, 成业 邬, 薇 程, 洲风 黄, 尊民 朱

**Affiliations:** 1 河南省人民医院，郑州大学人民医院，河南大学人民医院血液病研究所，郑州 450003 Hematological Institute of Henan Provincial People's Hospital, People's Hospital of Zhengzhou University, People's Hospital of Henan University, Zhengzhou 450003, China; 2 河南省人民医院检验科，郑州 450003 Laboratory of Henan Provincial People's Hospital, Zhengzhou 450003, China

多发性骨髓瘤（multiple myeloma, MM）是一种异质性很强的浆细胞克隆性增殖的恶性肿瘤，其预后分层对于MM的治疗有十分重要的意义。浆细胞的不成熟形态作为最早进行研究的预后因素之一，被证实与MM患者的预后关系密切。但国外学者多数采用Greipp等[1]提出的浆细胞分类，但其操作繁琐且不同中心一致性较差，临床应用较少。现总结河南省人民医院自2015年8月至 2020年3月收治的180例初诊MM患者的临床资料，根据我国浆细胞形态学标准，对每例患者250个骨髓有核细胞中原始幼稚浆细胞比例（bone marrow immature plasma cell percentage，BMIPCp）与MM实验室检查及预后的相关性进行研究，以探讨骨髓原始幼稚浆细胞负荷量能否成为简单有效的MM预后因素。

## 病例与方法

1. 病例：研究纳入我院自2015 年8月至 2020年3月收治的具备骨髓细胞形态学、血清学、流式细胞术结果及完整随访资料的180例初诊MM患者，诊断标准参考2020年中国多发性骨髓瘤诊治指南[2]。

2. 骨髓细胞形态分析：每例患者骨髓细胞形态学分析250个有核细胞，按照国内形态学标准，将浆细胞分为原始浆细胞、幼稚浆细胞（BMIPC）和成熟浆细胞[3]，计算BMIPCp与骨髓浆细胞比例（bone marrow plasma cell percentage，BMPCp）。

3. FISH检测：抽取患者抗凝骨髓液5～10 ml，经过CD138磁珠分选后进行浆细胞FISH检测，所有患者均进行del（17p）、del（13q）、1q21扩增、IGH重排初筛，部分患者继续检测了IGH重排方式，包括IGH/CCND1、IGH/FGFR3、IGH/MAF、IGH/MAFB（荧光探针为广州安必平医药科技有限公司产品），数目异常的阈值为20％，基因重排阈值为10％[4]，结果按照《人类细胞遗传学国际命名体制2016》的标准进行描述。

4. 化疗方案及疗效评价：180例患者中不包含接受自体造血干细胞移植、CAR-T细胞疗法、CD38单抗治疗的患者。采用以硼替佐米为基础的联合方案化疗4～8个周期，随后进入维持治疗。4个疗程的化疗结束后参照文献[2]进行疗效评估。疗效分为：严格意义上的完全缓解（sCR）、完全缓解（CR）、非常好的部分缓解（VGPR）、部分缓解（PR）、微小缓解（MR）、疾病稳定（SD）、疾病进展（PD）。客观缓解率（ORR）为sCR率、CR率、VGPR率、PR率的总和。

5. 随访：随访日期截至2020年7月30日。中位随访时间23.5（4.0～136.0）个月。总生存（OS）时间为自确诊之日至死亡或末次随访时间。无进展生存（PFS）时间为自确诊之日至疾病发生进展或死亡的时间。

6. 统计学处理：采用SPSS 25.0软件进行统计学分析，计量资料采用独立样本*t*检验或秩和检验，计数资料采用卡方检验，缓解率影响因素评估采用Logistic回归分析，Kaplan-Meier法评估单个危险因素的生存差异，多因素生存分析采用Cox风险模型，*P*<0.05为差异有统计学意义。

## 结果

1. 一般临床特征：180例患者中男101例，女79例，中位年龄58（37～80）岁。ISS分期Ⅰ或Ⅱ期患者104例（57.8％），Ⅲ期患者76例（42.2％）；BMPCp≥50％的患者38例（21.1％），BMPCp<50％的患者142例（78.9％）；IgG型患者80例（44.44％），IgA型55例（30.55％），IgD型18例（10.00％），IgM型3例（1.67％），轻链型21例（11.67％），不分泌型3例（1.67％）。

2. BMIPCp与临床特征的关系：根据180例患者BMIPCp的中位数，将MM患者分为BMIPCp≥18.3％组和BMIPCp<18.3％组，对两组患者的年龄、性别、ISS分期、BMPCp、β_2_-微球蛋白（β_2_-MG）、LDH、HGB、血清钙等指标进行比较（表1）。结果显示，BMIPCp≥18.3％组的ISS Ⅲ期患者比例、血清LDH含量、BMPCp显著高于BMIPCp<18.3％组（*P*值分别为0.037、0.001、<0.001）。

**表1:** BMIPCp<18.3％组与BMIPCp≥18.3％组多发性骨髓瘤患者临床特征比较

特征	BMIPCp<18.3％（120例）	BMIPCp≥18.3％（60例）	统计量	*P*值
年龄（岁，*x*±*s*）	58.18±0.86	59.82±1.24	*t*=−1.093	0.276
性别［例（％）］			*χ*^2^=2.689	0.100
男	69（57.5）	32（53.3）		
女	51（42.5）	28（46.7）		
ISS分期［例（％）］			*χ*^2^=4.356	0.037
Ⅰ/Ⅱ期	73（60.8）	31（51.7）		
Ⅲ期	47（39.2）	29（48.3）		
HGB［g/L，*M*（范围）］	85.0（44.0～140.0）	82.5（41.0～136.0）	*Z*=−1.083	0.279
血钙[mmol/L，*M*（范围）］	2.30（1.08～3.60）	2.37（1.69～3.49）	*Z*=−1.376	0.169
β_2_-微球蛋白［mg/L，*M*（范围）］	4.88（1.09～65.64）	5.20（1.08～83.63）	*Z*=−1.138	0.255
白蛋白（g/L，*x*±*s*）	33.38±0.60	31.96±0.83	*t*=1.384	0.168
乳酸脱氢酶［IU/L，*M*（范围）］	166.0（67.0～757.0）	203.5（96.0～738.0）	*Z*=−3.259	0.001
BMPCp［％，*M*（范围）］	24.0（1.6～78.0）	46.0（25.2～95.6）	*Z*=−7.179	<0.001
疗效［例（％）］			*χ*^2^=0.754	0.385
≥PR	93（82.3）	49（87.5）		
<PR	20（17.7）	7（12.5）		

注：BMIPCp：骨髓原始幼稚浆细胞比例；ISS：国际积分系统；BMPCp：骨髓浆细胞比例；PR：部分缓解

3. BMIPCp与遗传学异常的关系：BMIPCp≥18.3％组del（13q）的发生率明显高于BMIPCp<18.3％组（55.7％对32.0％，*χ*^2^＝8.826，*P*＝0.003），BMIPCp≥18.3％组1q21扩增的发生率明显高于BMIPCp<18.3％组（60.7％对44.0％，*χ*^2^＝4.204，*P*＝0.040），两组del（17p）、IGH重排的发生率差异无统计学意义（*P*值分别为0.238和0.086）。BMIPCp≥18.3％组无遗传学异常比例显著低于BMIPCp<18.3％组（4.9％对20.0％，*χ*^2^＝7.038，*P*＝0.012）。共66例患者进行了IGH/CCND1、IGH/FGFR3、IGH/MAF、IGH/MAFB检测，未发现两组间高危遗传学异常［包括del（17p）、IGH/FGFR3、IGH/MAF］发生率的差异有统计学意义（45.0％对28.6％，*χ*^2^＝1.633，*P*＝0.201）。

4. BMIPCp与治疗反应的关系：4个疗程化疗结束后评估疗效，采用*χ*^2^检验比较BMIPCp与疗效的关系。结果显示，BMIPCp≥18.3％组ORR为87.5％，BMIPCp<18.3％组ORR为82.3％，差异无统计学意义（*χ*^2^＝0.754，*P*＝0.385）。

5. 单因素生存分析：180例MM患者的中位PFS时间为16.0（95％*CI* 13.2～18.8）个月，中位 OS 时间为44.0（95％*CI* 33.7～54.3）个月。Kaplan-Meier生存分析显示，年龄≥60岁（*χ*^2^＝10.275，*P*＝0.001）、ISS Ⅲ期（*χ*^2^＝6.300，*P*＝0.012）、del（17p）（*χ*^2^＝4.808，*P*＝0.028）、BMPCp≥50％（*χ*^2^＝6.711，*P*＝0.010）、BMIPCp≥18.3％（*χ*^2^＝11.135，*P*＝0.001）对初诊MM患者的OS有显著影响（表2）。

**表2:** 影响初诊多发性骨髓瘤患者总生存的单因素和多因素分析结果

参数	单因素分析		多因素分析	
	*OR*（95％ *CI*）	*P*值	*OR*（95％ *CI*）	*P*值
年龄（≥60岁，<60岁）	2.180（1.333～3.565）	0.001	1.905（1.154～3.142）	0.012
β_2_-微球蛋白（≥3.5mg/L，<3.5mg/L）	1.479（0.912～2.400）	0.108	−	−
BMPCp（≥50％，<50％）	2.125（1.181～3.823）	0.010	1.873（0.901～3.895）	0.093
BMIPCp（≥18.3％，<18.3％）	2.346（1.395～3.944）	0.001	2.352（1.243～4.449）	0.009
ISS分期（Ⅲ期，Ⅰ、Ⅱ期）	1.864（1.134～3.065）	0.012	1.686（1.020～2.787）	0.042
LDH（≥250 IU/L，<250 IU/L）	1.443（0.855～2.436）	0.165	1.312（0.760～2.265）	0.329
是否伴del（17p）（是，否）	2.410（1.067～5.444）	0.028	1.525（0.657～3.539）	0.326
是否伴1q21扩增（是，否）	1.231（0.694～2.183）	0.474	−	−

注：BMIPCp：骨髓原始幼稚浆细胞比例；BMPCp：骨髓浆细胞比例；−：未进行多因素分析

BMIPCp≥18.3％组与BMIPCp<18.3％组中位OS时间分别为30.0（95％*CI* 24.0～36.0）个月和48.0（95％*CI* 37.6～58.4）个月，差异有统计学意义（*χ*^2^＝11.135，*P*＝0.001）（图1）。BMIPCp≥18.3％且BMPCp≥50％组OS时间最短（中位OS时间17.0个月，95％*CI* 14.6～19.4个月），与BMPCp≥50％且BMIPCp<18.3％组（中位OS时间未达到）、BMPCp<50％且BMIPCp≥18.3％组（中位OS时间33.0个月，95％*CI* 24.7～41.3个月）、BMPCp<50％且BMIPCp<18.3％组（中位OS时间48.0个月，95％*CI* 37.8～58.2个月）相比，差异均有统计学意义（*χ*^2^值分别为3.899、6.187和20.663，*P*值分别为0.048、0.013和<0.001）（图2）。

**图1:**
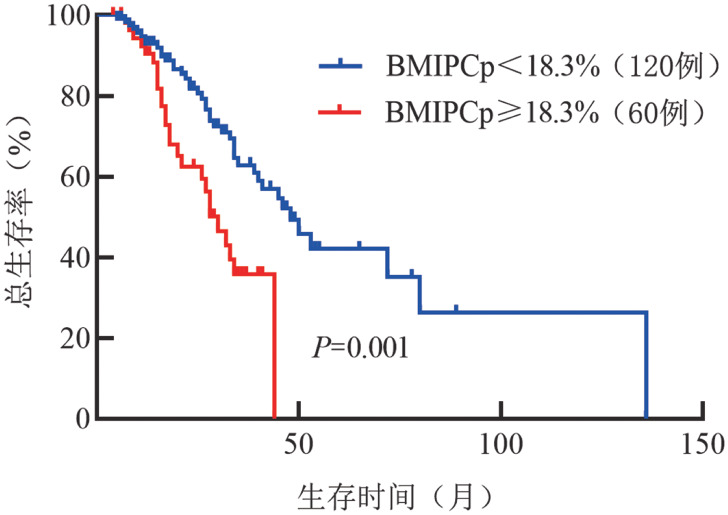
BMIPCp≥18.3％组与BMIPCp<18.3％组多发性骨髓瘤患者的总生存曲线 BMIPCp：骨髓原始幼稚浆细胞比例

**图2:**
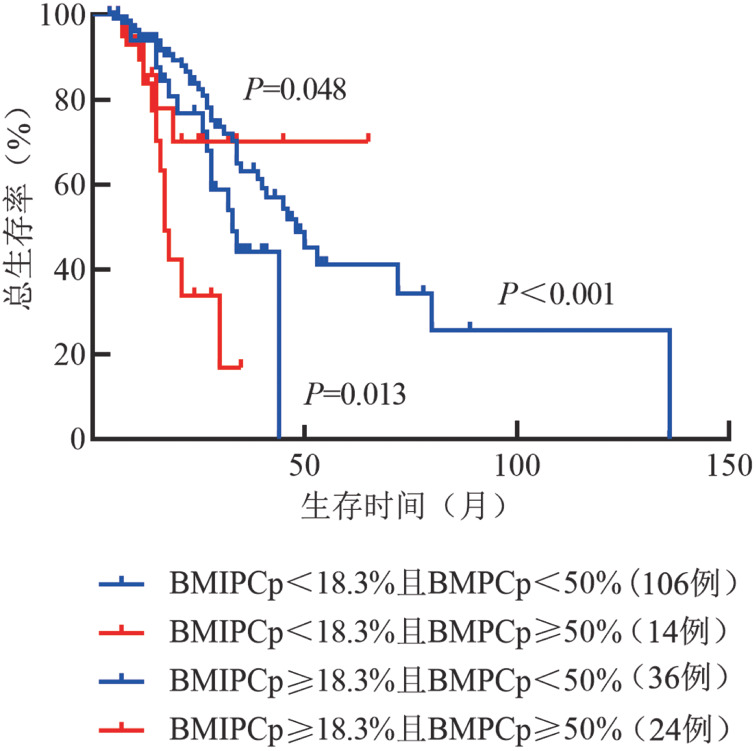
BMPCp≥50％且BMIPCp≥18.3％组、BMPCp≥50％且BMIPCp<18.3％组、BMPCp<50％且BMIPCp≥18.3％组、BMPCp<50％且BMIPCp<18.3％组多发性骨髓瘤患者的总生存曲线 BMIPCp：骨髓原始幼稚浆细胞比例；BMPCp：骨髓浆细胞比例；*P*值为各组与BMPCp≥50％且BMIPCp≥18.3％组比较

BMIPCp≥18.3％组与BMIPCp<18.3％组中位PFS时间分别为13.0（95％*CI* 8.4～17.6）个月和21.0（95％*CI* 15.8～26.2）个月，差异无统计学意义（*χ*^2^＝2.580，*P*＝0.108）。仅BMIPCp≥18.3％且BMPCP≥50％组与BMIPCp<18.3％且BMPCP<50％组PFS的差异有统计学意义（14.0个月对22.0个月，*χ*^2^＝3.894，*P*＝0.048）（图3）。

**图3:**
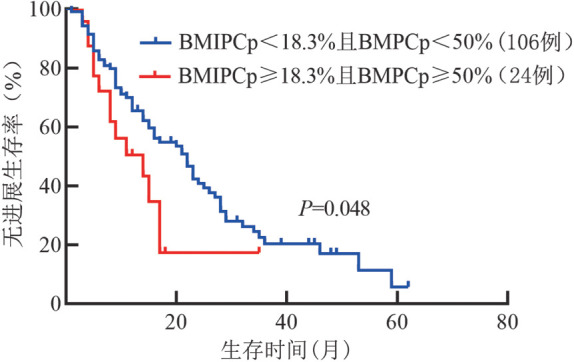
BMPCp≥50％且BMIPCp≥18.3％组与BMPCp<50％且BMIPCp<18.3％组的无进展生存曲线 BMIPCp：骨髓原始幼稚浆细胞比例；BMPCp：骨髓浆细胞比例

6. 多因素生存分析：进一步将上述变量纳入Cox风险回归模型进行多因素分析，结果显示，年龄≥60岁（*OR*＝1.905，95％*CI* 1.154～3.142，*P*＝0.012）、BMIPCp≥18.3％（*OR*＝2.352，95％*CI* 1.243～4.449, *P*＝0.009）、ISS Ⅲ期（*OR*＝1.686，95％*CI* 0.760～2.265，*P*＝0.042）是初诊MM患者OS的独立危险因素（表2）。未发现影响患者PFS的独立危险因素（表3）。

**表3:** 影响初诊多发性骨髓瘤患者无进展生存的单因素和多因素分析结果

参数	单因素分析		多因素分析	
	*OR*（95%*CI*）	*P*值	*OR*（95%*CI*）	*P*值
年龄（≥60岁，<60岁）	1.189（0.828～1.707）	0.474	–	–
β_2_-微球蛋白（≥3.5 mg/L，<3.5 mg/L）	1.238（0.860～1.783）	0.239	–	–
BMPCp（≥50％，<50％）	1.408（0.902～2.197）	0.295	–	–
BMIPCp（≥18.3％，<18.3％）	1.364（0.924～2.014）	0.108	1.112（0.719～1.722）	0.633
ISS分期（Ⅲ期，Ⅰ、Ⅱ期）	1.298（0.900～1.872）	0.152	1.453（0.961～2.196）	0.076
LDH（≥250 IU/L，<250 IU/L）	1.236（0.827～1.847）	0.289	1.122（0.720～1.749）	0.610
是否伴del（17p）（是，否）	2.127（1.091～4.149）	0.021	1.629（0.811～3.272）	0.107
是否伴1q21扩增（是，否）	1.304（0.858～1.981）	0.204		

注：BMIPCp：骨髓原始幼稚浆细胞比例；BMPCp：骨髓浆细胞比例；–：未进行多因素分析

## 讨论

1985年Greipp等[1]提出，根据MM患者骨髓细胞形态学中浆细胞核染色质是否出现聚集、是否出现清晰的核仁、核仁大小、核质比，把浆细胞分为浆母细胞（plasmablast，PB）、不成熟浆细胞、中间型浆细胞和成熟浆细胞。计数500个浆细胞，其中PB≥2％的MM是一类预后很差的亚型，定义为浆母细胞型骨髓瘤（plasmablastic multiple myeloma，PBMM）。该类患者浆细胞标记指数增高，血清IL-6受体增高，在骨髓活检组织中表现为更高的微血管密度和Ki-67、血管内皮生长因子表达，同时具有更高的浆细胞负荷和疾病分期[5]–[8]。Rajkumar等[9]发现，即使经过自体造血干细胞移植，PB组的预后依然较非PB组差（中位OS时间5个月对24个月，中位PFS时间4个月对12个月，*P*值均<0.001）。之后的多项研究也证实MM患者骨髓浆细胞成熟度与预后关系密切[10]–[12]。

但PB≥2％却始终未有效地应用于临床以指导患者的预后分层，原因如下：①Greipp等[1]的浆细胞分类系统要求额外分析500个浆细胞，工作量大；②2％的阈值以及区分PB、幼稚型浆细胞、中间型浆细胞的复杂标准造成不同中心的PBMM患者比例差异较大。Greipp等[5]在453例MM患者中发现8.2％具有≥2％的PB；Rajkumar等[9]的研究中其比例为28％；Al-Sahmani等[13]在139例患者中未发现PB。我国浆细胞形态学标准为成熟浆细胞胞体较小，常有核旁淡染区，染色质呈块状，无核仁，与原始幼稚浆细胞的形态学差异明显，结果具有较高重复性，计数全骨髓有核细胞中原始幼稚浆细胞比例的同时兼顾了浆细胞负荷，且有研究证明，将PB和幼稚浆细胞作为一组时同样具有显著的预后意义[14]–[15]。我们的研究显示，BMIPCp≥18.3％组ISS Ⅲ期患者比例、LDH、BMPCp明显高于BMIPCp<18.3％组，说明该组患者肿瘤负荷高，与疾病分期高相关。

1q21扩增是MM的常见遗传学异常，虽未被纳入R-ISS的高危遗传学异常，但将1q21扩增作为MM的独立不良预后因素并无争议[16]–[18]。FISH检测到del（13q）也是MM的不良预后因素，在多项研究的单因素分析中，del（13q）阳性MM患者的预后明显较差[19]–[20]。FISH未检测到遗传学异常是MM的低危因素，Boyd等[21]的报道显示，该类患者的中位PFS和OS时间分别为23.5和60.6个月，明显优于具有>1种高危遗传学异常的患者（中位PFS时间11.7个月，中位OS时间21.7个月），差异有统计学意义（*P*值均<0.001）。本研究中，BMIPCp≥18.3％组1q21扩增、del（13q）的发生率明显高于BMIPCp<18.3％组，无遗传学异常的患者比例明显较低，提示BMIPCp≥18.3％患者遗传学高危。

本研究中的患者均接受以硼替佐米为基础的联合方案，BMIPCp≥18.3％与BMIPCp<18.3％组患者的ORR差异无统计学意义，均达到80％以上，PFS时间差异无统计学意义（13.0个月对21.0个月，*χ*^2^＝2.580，*P*＝0.108），与Takakuwa等[22]的结果不同。Takakuwa等[22]报道了30例以硼替佐米或来那度胺为基础治疗的MM患者，幼稚浆细胞≥1％者的PFS时间更短（6.2个月对未达到，*P*＝0.001）。造成这种差异的原因可能是该研究病例数较少。BMIPCp≥18.3％组与BMIPCp<18.3％组OS时间的差异有统计学意义（30.0个月对48.0个月，*χ*^2^＝11.135，*P*＝0.001），且BMIPCp≥18.3％是MM预后不良的独立影响因素。

浆细胞负荷是MM患者的重要预后因素，我们的研究发现，BMPCp≥50％的MM患者与BMPCp<50％患者的OS具有显著差异，与Al Saleh等[23]和Hwang等[24]的结果相符。BMPCp≥50％且BMIPCp≥18.3％组与BMPCp≥50％且BMIPCp<18.3％组相比，OS时间明显缩短。BMIPCp<18.3％且BMPCp<50％组是预后最好的一组，仅BMIPCp≥18.3％且BMPCp≥50％组与BMIPCp<18.3％且BMPCp<50％组PFS的差异有统计学意义。我们认为，以硼替佐米为基础的联合方案能对成熟及幼稚浆细胞起到有效的杀伤作用[25]，可改善部分BMIPCp≥18.3％组患者的PFS，但不能纠正OS[26]。

尽管浆细胞形态对预后的影响未被纳入目前的MM预后评估体系，但MM的诊断需要骨髓细胞形态学分析，骨髓原始幼稚浆细胞负荷量可以同步获得，较为简便，重复性较高，兼顾浆细胞不成熟形态和浆细胞负荷，且与MM患者的高危遗传学异常密切相关。我们的结果显示BMIPCp≥18.3％是MM患者不良预后的独立影响因素，与BMPCp结合可以更精准地判断MM患者的危险分层，适合作为初诊MM患者的预后评估指标。
